# Trends in Volume, Timeliness, and Reporting Quality of Oncology Systematic and Scoping Reviews: A 25-Year Analysis

**DOI:** 10.3390/curroncol33090571

**Published:** 2026-09-21

**Authors:** Nandi Edwards, Hadia Malik, Daniel Zhang, Gabriel Boldt, Michael Lock, Jennifer Leigh, Ronald Chow

**Affiliations:** 1 Sunnybrook Health Sciences Centre, Temerty Faculty of Medicine, University of Toronto, Toronto, ON M4N 3M5, Canada; 2 Siebel School of Computing and Data Science, University of Illinois Urbana-Champaign, Urbana, IL 61801, USA; 3 London Health Sciences Centre, Schulich School of Medicine & Dentistry, University of Western Ontario, London, ON N6A 5W9, Canada; 4 Centre for Evidence-Based Medicine, University of Oxford, Oxford OX2 6GG, UK

**Keywords:** systematic review, scoping review, oncology, protocol registration, PROSPERO, publication lag, PRISMA, evidence synthesis

## Abstract

Systematic and scoping reviews synthesize evidence in oncology’s fast-moving therapeutic landscape, informing clinical and policy decisions. However, little is known about how review volume has changed over time, how quickly reviews reach publication, or how often protocol registration is reported within review abstracts. We searched Medline (PubMed), Embase, and the Cochrane Library (2000 to 9 October 2025) for oncology-focused articles labelled as systematic or scoping reviews, screened in duplicate by a human reviewer and a ChatGPT-5-assisted system, with a third reviewer resolving disagreements. Cancer subspecialty, search-to-publication interval, and abstract-level protocol registration status and registry type were extracted. Of 92,800 references, 49,378 (53%) were systematic (*n* = 46,318) or scoping (*n* = 3060) reviews. Review volume rose sharply from 2000 (*n* = 61) to 2024 (*n* = 6313), with scoping reviews increasing steadily. Data for 2025 were available only through 9 October and were therefore not directly comparable with prior complete years. Among 9297 reviews reporting search dates, mean search-to-publication lag was 13 months, unchanged over time (*p* = 0.09). Only 2235 reviews (4.5%) reported protocol registration in their abstract, predominantly via the International Prospective Register of Systematic Reviews (PROSPERO). Oncology evidence-synthesis output has grown, yet publication timeliness and abstract-level registration reporting have not improved, raising questions about its currency and verifiability.

## Simple Summary

Clinicians and policymakers rely on review articles that gather and summarize cancer research to keep up with a fast-moving field. We examined 25 years of these reviews to understand how many are published each year, how long they take to appear after the literature is searched, and how often their abstracts report that a study plan was registered in advance. We found that the number of cancer reviews has grown substantially, with increasing use of scoping reviews, which broadly map the available evidence on a topic. Despite this growth, reviews still take about a year to reach publication after the literature is searched, and fewer than 1 in 20 note in their abstract that their plan was registered. As the number of reviews continues to grow, more timely publication and transparent reporting could help ensure that the evidence guiding cancer care remains current and can be readily evaluated.

## 1. Introduction

Systematic and scoping reviews play an important role in oncology by bringing together a rapidly expanding evidence base, identifying gaps, and informing clinical guidelines and policy decisions. Both types of reviews have become increasingly common, but they serve distinct purposes. Systematic reviews typically address focused questions using predefined eligibility criteria and formal appraisal of study quality, whereas scoping reviews are used to map the breadth and characteristics of the available literature, identify gaps, and generally do not include formal quality assessment [1].

As the volume of oncology research grows, however, producing a review is only useful if its findings remain current. Even when a review is published, there is typically a time lag between the date of the literature search and the date of publication [2]. Given how rapidly oncology literature evolves, new evidence may emerge by the time findings reach the readers [3]. Despite this, the search-to-publication lag of oncology systematic and scoping reviews, and whether it has improved over time, has not been well characterized.

Transparency in how reviews are planned and conducted is equally important. Over the past decade, increasing emphasis has been placed on publicly registering review protocols prior to study initiation to reduce selective reporting and duplication [4]. By establishing the intended methods before a review begins, protocols also make deviations easier to identify [4]. This shift is reflected in the evolution of the Preferred Reporting Items for Systematic Reviews and Meta-Analyses (PRISMA) guidelines [4,5,6]. PRISMA 2009 established foundational reporting standards, followed by PRISMA for Protocols (PRISMA-P) 2015, PRISMA Extension for Scoping Reviews (PRISMA-ScR) 2018, and PRISMA 2020, which emphasized transparent reporting, including protocol registration in abstracts [4,5,6]. Despite these developments, uptake remains limited across journals of varying impact, highlighting a persistent gap between recommended practice and implementation [7].

Oncology reviews often synthesize evidence across heterogeneous trials, including rapidly evolving therapeutic classes, to inform clinical decision-making and guideline development [8]. Differences in study selection, analytic decisions, or outcome reporting can shift pooled estimates and perceived benefit–risk profiles, while delays in publication can leave those estimates behind the current evidence base. Timely publication and transparent reporting are therefore central to how reliably oncology reviews can inform practice.

In this study, we document the annual number of oncology systematic and scoping reviews published over the past 25 years. We quantified annual publication volume and the interval between literature search and publication, and evaluated how often reviews reported protocol registration in their abstracts and how this practice has changed over time.

## 2. Materials and Methods

Comprehensive searches were developed with an information specialist (G.B.) and conducted in Medline (PubMed), Embase, and the Cochrane Library from 2000 to 9 October 2025. The search concepts were cancer and review articles (Supplementary File S1).

Search results were screened to identify which articles explicitly declared their work to be either a systematic review or scoping review in the title or abstract. Articles that did not declare were excluded. This inclusion criterion allowed for a consistent classification across the study period without inferring methodology from broader terminology such as “evidence synthesis” or “review”, which may encompass non-systematic approaches. It also aligns with PRISMA guidance recommending that authors identify the review type in the title [5,6].

Of the included articles, we reviewed the abstracts to determine the author-declared cancer subspecialty (medical oncology, radiation oncology, surgical oncology, supportive oncology, or multiple subspecialties) and computed the time in months between search and publication. Because PRISMA 2020 lists protocol registration as an abstract reporting item, and because abstracts are openly indexed while full texts often are not, we assessed each abstract for any mention of protocol registration, recording registry type as International Prospective Register of Systematic Reviews (PROSPERO), Open Science Framework (OSF), or Other.

All search results and extraction were screened in duplicate by one human reviewer (R.C.) and one heuristic-assisted reviewer using ChatGPT-5 (OpenAI, San Francisco, CA, USA) (D.Z.). The LLM returned extracted data in JSON format, along with a corresponding reasoning summary and confidence score (low, medium, high). Discrepancies were resolved by discussion, with ChatGPT-5 prompted to provide reasoning behind its screening decisions. Where consensus could not be reached, a third reviewer (J.L.) assisted in adjudication.

Annual trends in the number of reviews and the mean search-to-publication interval were plotted overall and by cancer subspecialty. Linear regression assessed temporal trends in this interval, with Type I error set at 0.05. Reporting patterns were summarized using descriptive statistics, and annual proportions of reviews reporting protocol registration and the distribution of registry types were calculated to assess trends over time. All analyses were conducted using StataBE 18.0.

Generative AI was used solely as a screening aid. ChatGPT-5 (OpenAI, San Francisco, CA, USA) was not used to generate, analyze, or interpret data, or to draft the manuscript.

## 3. Results

A total of 92,800 unique references were identified. Of these, 49,378 (53%) were explicitly declared as a systematic or scoping review in the title or abstract, a systematic-to-scoping ratio of approximately 15:1 (46,318 systematic and 3060 scoping reviews). The most common subspecialty classifications were multiple oncology disciplines (10,204; 21%), supportive oncology (7618; 15%), surgical oncology (5184; 11%), medical oncology (5070; 10%), and radiation oncology (1290; 3%). Of all systematic and scoping reviews, 2235 (4.5%) reported protocol registration in their abstract.

**Figure 1:**
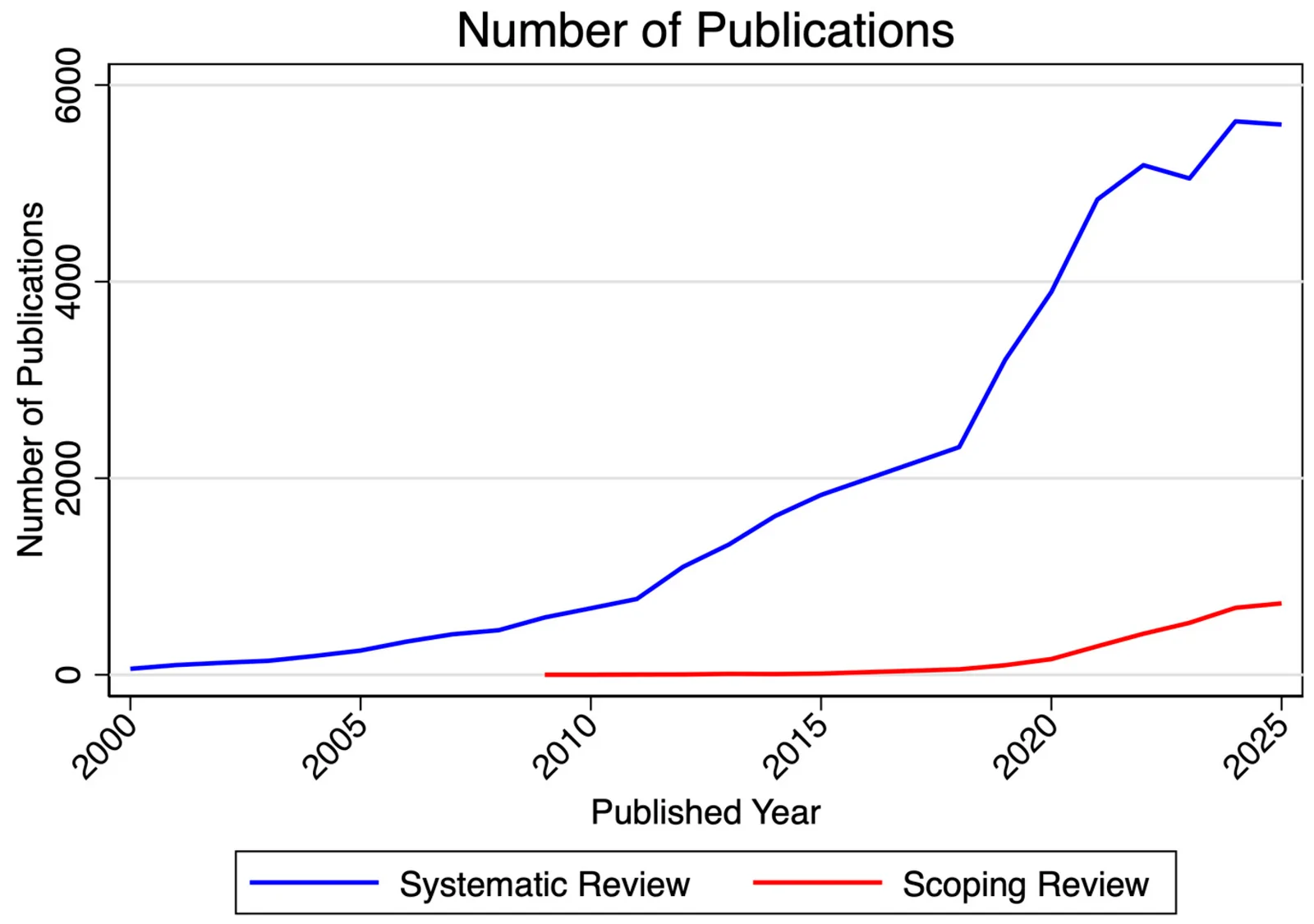
Annual number of oncology systematic and scoping reviews published from 2000 to 2025. Blue, systematic reviews; red, scoping reviews. The x-axis indicates publication year and the y-axis the number of publications.

The number of reviews increased steadily from 2000 (*n* = 61) to 2024 (*n* = 6313) (Figure 1). Data for 2025 were incomplete, reflecting only publications indexed through 9 October, and therefore were not directly comparable with earlier complete calendar years. Within this partial window, 5598 systematic and 727 scoping reviews were recorded, compared with 5631 and 682, respectively, across all of 2024. Because these totals span roughly nine months against a full prior year, they should not be read as a within-year decline in systematic reviews. This rising proportion of scoping reviews over the study period was consistent across all cancer subspecialties (Figure S1).

**Figure 2:**
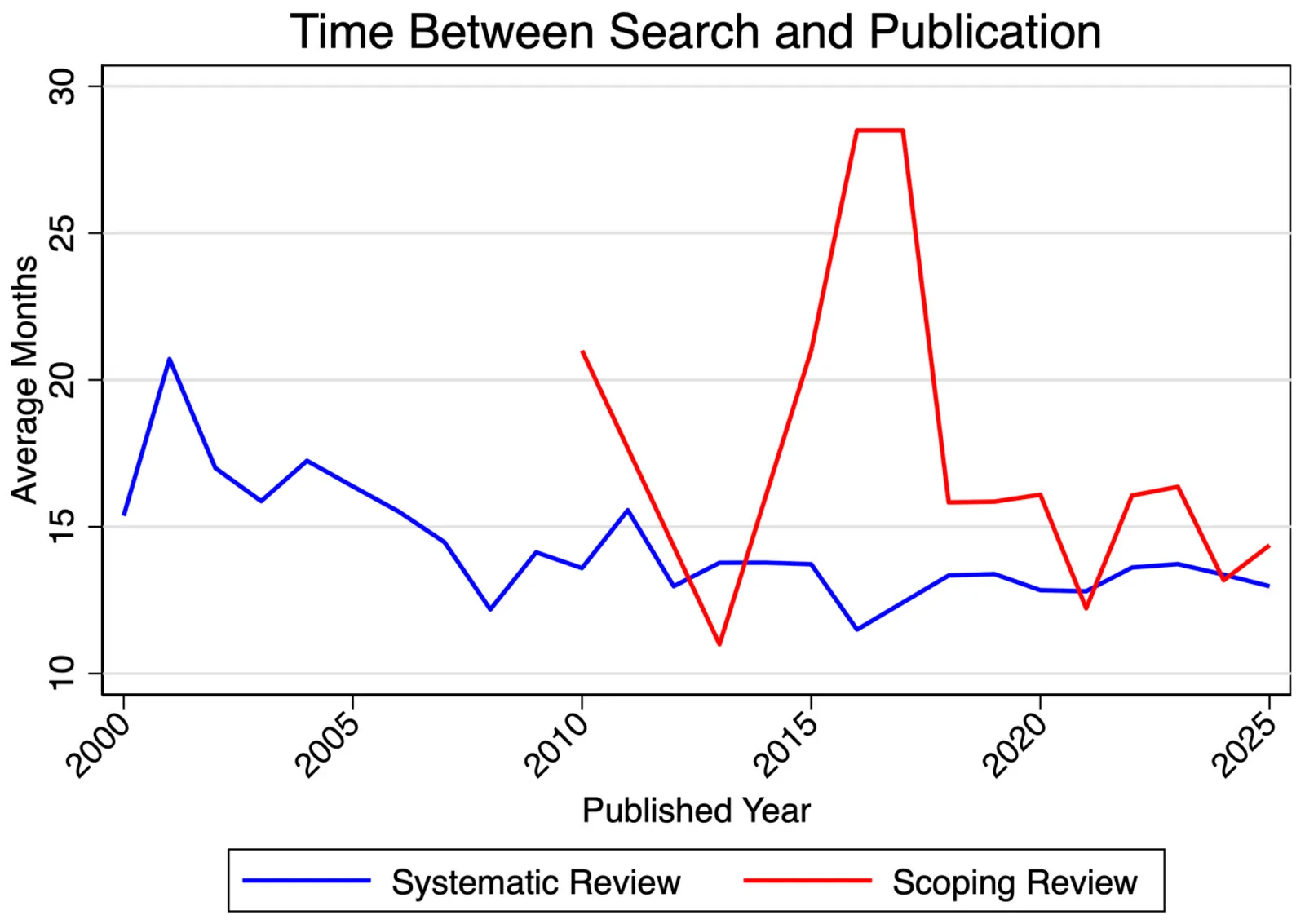
Mean interval (months) between literature search and publication for oncology systematic and scoping reviews, by publication year, 2000–2025. Blue, systematic reviews; red, scoping reviews. The x-axis indicates publication year and the y-axis the mean interval in months.

Among 9297 reviews reporting a search date in the abstract, the mean interval between the literature search and publication was 13 months (Figure 2). This lag did not differ by year (*p* = 0.09) or by review type and was consistent across all cancer subspecialties (Figure S2).

**Figure 3:**
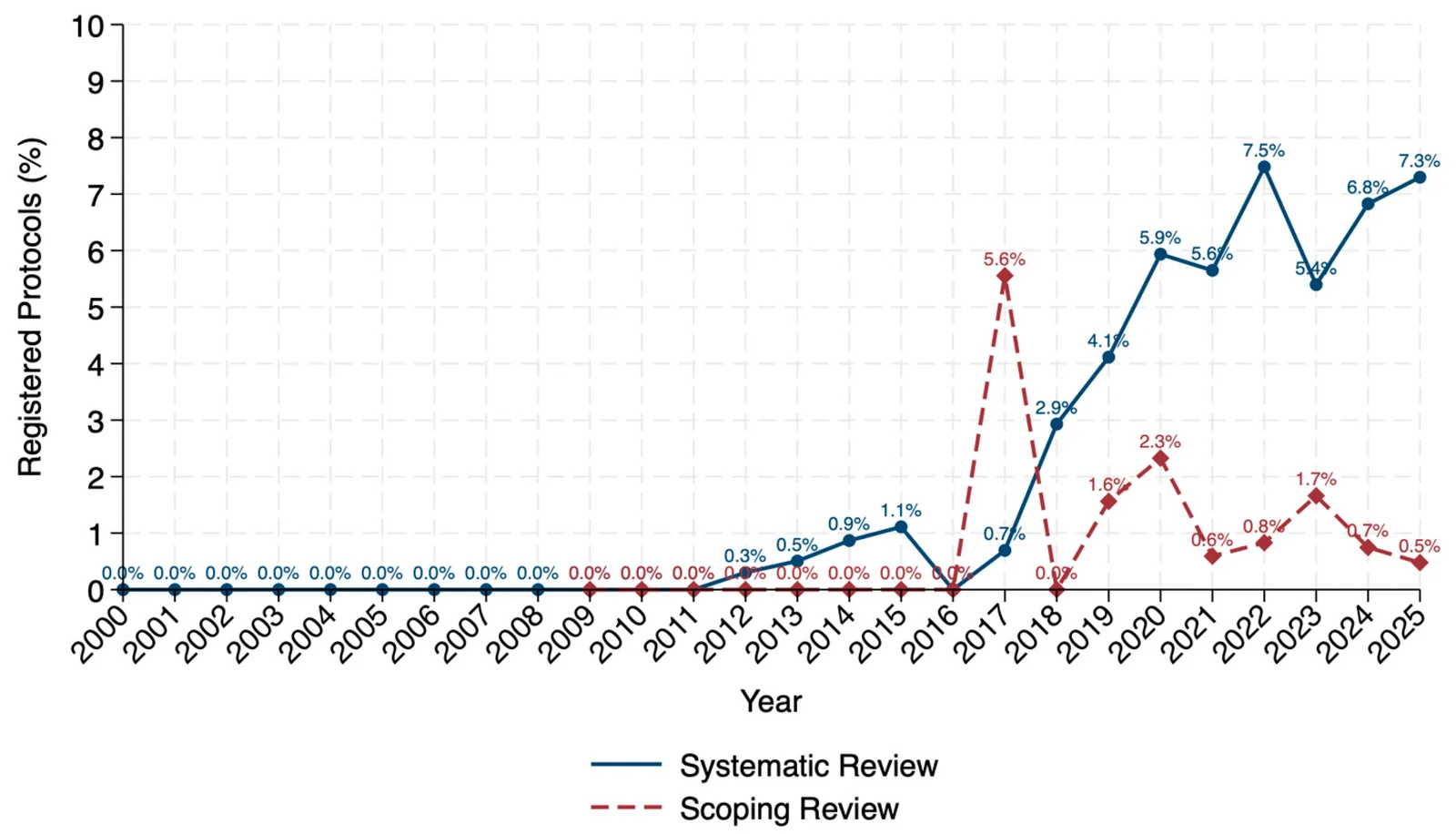
Annual percentage of oncology systematic and scoping reviews reporting protocol registration in their abstracts, from 2000 to 2025. Solid blue, systematic reviews; dashed red, scoping reviews. The x-axis indicates publication year and the y-axis the percentage of reviews reporting protocol registration.

Abstract-level reporting of protocol registration was negligible across both review types from 2000 through 2011, with only minimal uptake through 2016 (≤1.1%) (Figure 3). From 2018, systematic review reporting rose from 2.9% to a peak of 7.5% in 2022, then remained between 5.4% and 7.3% through 2025. Scoping reviews showed lower and more variable reporting, with a brief peak in 2017 (5.6%) but remaining below 2% from 2021 to 2025. Overall, abstract-level protocol reporting stayed below 8% throughout the study period.

In absolute terms, the number of reviews reporting protocol registration in the abstract increased from 108 before 2019 to 401 in 2025, with the largest year-over-year rise between 2021 and 2022 (230 to 352) (Table 1). Despite this growth, the proportion reporting registration remained low. Among reviews reporting registration, PROSPERO accounted for the majority across all years (81%–100%). OSF first appeared in 2020 (15%) before declining to 1%–2% in subsequent years, while other registries remained uncommon (≤1%–4% annually).

## 4. Discussion

To our knowledge, this is the first study in oncology to assess long-term trends in the volume and distribution of systematic and scoping reviews alongside their search-to-publication lag and abstract-level reporting of protocol registration. Over the past 25 years, the volume of evidence synthesis in oncology has grown substantially. Systematic reviews have historically increased year over year, while scoping reviews have risen steadily over the past five years. Trends in 2025 should be interpreted cautiously given the partial year of data. Despite the different purposes and methodological requirements of these review types, both had a similar search-to-publication lag of approximately one year, with no meaningful improvement over time.

The diverging trajectories of systematic and scoping reviews may reflect a combination of methodological, pragmatic, and structural factors. Conducting a systematic review has become increasingly formalized, with expectations of protocol registration, comprehensive risk-of-bias assessments, and adherence to reporting standards. Although these processes improve methodological rigour and transparency, they also require considerable time, expertise, and resources [9]. In contrast, scoping reviews, which are designed to map evidence and identify gaps, do not typically require critical appraisal and may offer more flexibility for broad or heterogeneous research questions. Their increasing use in oncology may therefore partly reflect the practical demands of contemporary evidence synthesis, particularly as the volume and complexity of the literature continue to grow.

At the same time, the rise in scoping reviews should not be interpreted solely as a response to the burden of systematic reviews. It may also reflect greater recognition and appropriate use of scoping review methodology for questions aimed at characterizing the breadth, nature, and gaps of an evidence base [10,11]. This distinction is important because the two designs serve different purposes, and growth in scoping reviews does not necessarily represent substitution for systematic reviews. Our findings describe the changing distribution of review types but cannot determine what is driving that change.

Timeliness presents a separate challenge. The mean 13-month lag did not improve over the study period, raising concerns that findings may be partly outdated by the time they are published, particularly in fast-moving areas such as drug development and precision oncology [12]. Importantly, this interval extends beyond the review itself, accumulating through peer review and revision cycles, author resubmission, editorial and production backlogs, and delays in database indexing [13]. Because we measured only the total search-to-publication interval, the lag cannot be attributed to any single stage or cause, including methodological demands of the review itself [12,14].

A parallel gap emerged when examining abstract-level reporting of protocol registration. Across the full study period, the observed rate was 4.5%. Reporting among systematic reviews increased following the introduction of more explicit reporting guidance, reaching 7.5% in 2022, but this increase was not sustained. The timing is consistent with greater attention to registration following PRISMA 2020, although our data cannot establish that the guideline itself caused the increase.

Among abstracts reporting registration, PROSPERO accounted for the vast majority of registrations, likely reflecting its early establishment in 2011, free access, rapid processing, and endorsement by major guidelines [15,16,17]. In contrast, OSF adoption remained limited despite its introduction in 2019, suggesting preference for a dedicated systematic review registry [15].

Notably, low abstract-level reporting may be due to several factors, including lack of reporting of registered protocols in abstracts and true non-registration. Registration gaps could reflect structural and behavioural barriers rather than lack of awareness or usability. While familiarity with protocol registration is high, previous work demonstrates that many authors do not register due to non-mandatory policies, limited institutional expectations, perceived administrative burden, and concerns about idea theft [17,18].

The comparatively lower abstract-level reporting rates observed in scoping reviews may relate to guideline differences. PRISMA 2020 includes protocol registration in its abstract checklist, whereas PRISMA-ScR lists it as optional and provides no abstract-specific guidance [5,6]. Meta-research in other fields similarly identifies protocol registration as one of the most frequently omitted reporting items in scoping reviews [19]. As scoping reviews become more common in oncology, greater consistency in how protocol information is reported may improve transparency standards across review types.

These reporting gaps matter because a prospectively specified protocol provides a reference against which changes in eligibility criteria, outcomes, and analytic methods can be assessed [20]. Concerns about methodological transparency are also relevant to reviews used directly in clinical guidance. Among reviews cited in National Comprehensive Cancer Network guidelines, only 50% adhered to PRISMA and 4.8% were rated as having low risk of bias, with protocol and eligibility criteria identified as the highest-risk domains [21,22]. Combined with the low visibility of protocol registration and a mean 13-month publication lag observed in our study, these findings highlight challenges in evaluating both the rigour and currency of oncology evidence syntheses.

A few practical measures could help narrow the gap between recommended standards and current practice. Firstly, protocol registration and transparency are one area for improvement. Journals and funders could make protocol registration an expectation rather than an encouraged practice, with registration status and the protocol identifier reported in a standardized field within the abstract. This is supported by evidence that prospectively registered reviews have been associated with greater methodological soundness [23], while uptake remains limited, in part, because of low awareness and the lack of a clear expectation to register [17]. Timeliness also needs greater attention. Search dates are often underreported, with one analysis of 300 systematic reviews finding that fewer than half stated the last search date in the abstract [14]. Journals could therefore require authors to report this date and prompt a search update before final acceptance, helping reduce the lag between evidence retrieval and publication. Additionally, keeping reviews current as evidence continues to emerge is a related challenge. In rapidly evolving areas of oncology, wider adoption of living systematic reviews, already used to support living oncology guidelines, could offer a more sustained way to keep conclusions current as new evidence emerges [24,25,26]. Finally, reducing the workload required to produce and maintain reviews may also help. Automated evidence-monitoring tools and responsible use of AI-assisted screening could lessen some of this burden, provided these tools are appropriately validated, and their use is transparently reported [27,28].

Several limitations should be considered. First, our analysis was restricted to abstract-level reporting. Reviews with a registered protocol that did not mention registration in the abstract were therefore not captured, meaning our estimates represent a lower bound on true registration. We also recorded registration only as present or absent and did not assess whether registered protocols were followed through to publication, meaning that reporting of registration should not be interpreted as evidence of methodological compliance. Reliance on author self-report introduces variability, as registration may be described differently across journals and disciplines. Our requirement that reviews explicitly identify themselves as systematic or scoping in the title or abstract may also have underestimated publication volume, particularly in earlier years when terminology and reporting practices were less standardized. If earlier reviews were less consistently labelled, the apparent growth in review volume, including the relative growth of scoping reviews, may be overestimated. We also did not assess the methodological quality or risk of bias of the included reviews, focusing instead on describing patterns in research output and reporting. Cancer subspecialties were assigned from author descriptions in the abstract and may have been misclassified when a subspecialty was not explicitly stated or a review crossed multiple disciplines. The search-to-publication analysis was also restricted to the 9297 of 49,378 reviews (18.8%) reporting a search date in the abstract, so the observed 13-month interval may not be representative of all included reviews. In addition, the search was limited to English-language abstracts, leaving evidence from other languages and regions underrepresented. Finally, variation in database indexing delays means that some recently published reviews may not yet have been captured, which could contribute to lower counts in the most recent period.

## 5. Conclusions

Over the past 25 years, oncology evidence synthesis has grown substantially, yet its timeliness and transparent reporting have not kept pace. Systematic and scoping reviews continue to face an approximately one-year lag between literature search and publication, while fewer than 5% report protocol registration in their abstracts. These findings highlight a persistent gap between recommended methodological standards and current practice. Closing this gap will require coordinated efforts across journals, funders, and institutions to strengthen transparency, improve timeliness, keep evidence syntheses current, and reduce the practical burden of producing and maintaining high-quality reviews.

## Figures and Tables

**Table 1:** Registries used for protocol registration. PROSPERO, International Prospective Register of Systematic Reviews; OSF, Open Science Framework; Other includes registries or repositories not classified as PROSPERO or OSF. Registry type was specified for 2040 of the 2235 reviews reporting protocol registration.

Publication Year	Total Reported	PROSPERO	OSF	Other
**≤2018**	108	105 (97%)	0 (0%)	3 (3%)
**2019**	118	118 (100%)	0 (0%)	0 (0%)
**2020**	187	152 (81%)	28 (15%)	7 (4%)
**2021**	230	201 (87%)	24 (10%)	5 (2%)
**2022**	352	343 (97%)	7 (2%)	2 (<1%)
**2023**	283	276 (98%)	6 (2%)	1 (<1%)
**2024**	361	353 (98%)	7 (2%)	1 (<1%)
**2025**	401	392 (98%)	6 (1%)	3 (1%)
**Total**	**2040**	**1940 (95%)**	**78 (4%)**	**22 (1%)**

## Data Availability

All data generated or analysed during this study are included in this article and its Supplementary Materials.

## Supplementary Materials

The following supporting information can be downloaded at: https://www.mdpi.com/article/10.3390/curroncol33090571/s1. Figure S1: Number of Publications Over Time by Review Type, by Cancer Subspecialty; Figure S2: Average Time (Months) Between Search and Publication by Review Type, by Cancer Subspecialty; File S1: Search Strategies.

## Abbreviations

The following abbreviations are used in this manuscript:

PRISMA	Preferred Reporting Items for Systematic Reviews and Meta-Analyses
PRISMA-P	PRISMA for Protocols
PRISMA-ScR	PRISMA Extension for Scoping Reviews
PROSPERO	International Prospective Register of Systematic Reviews
OSF	Open Science Framework
