# Supplementary material for: Trends in Volume, Timeliness, and Reporting Quality of Oncology Systematic and Scoping Reviews: A 25-Year Analysis
Source: Curr Oncol. 2026 Sep 21;33(9):571. doi: 10.3390/curroncol33090571 (PMC13604965; doi:10.3390/curroncol33090571)
Supplement: Supplementary file 1 [file curroncol-33-00571-s001.zip › Supplementary Figures.pdf]

Figure S1. Number of Publications Over Time by Review Type, within **1.1** Medical Oncology **1.2** Radiation Oncology **1.3** Surgical Oncology **1.4** Supportive Oncology **1.5** Multiple Subspecialties

1.1

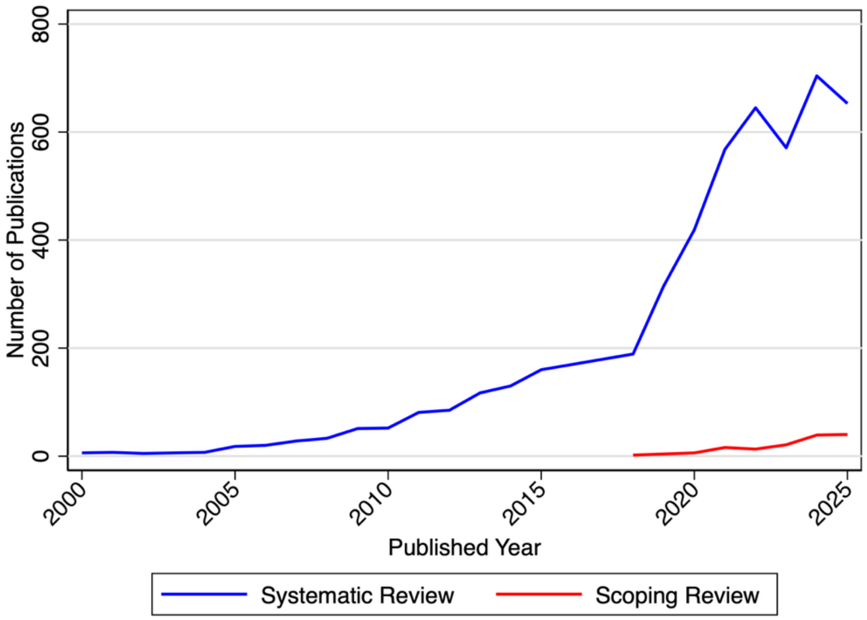

1.2

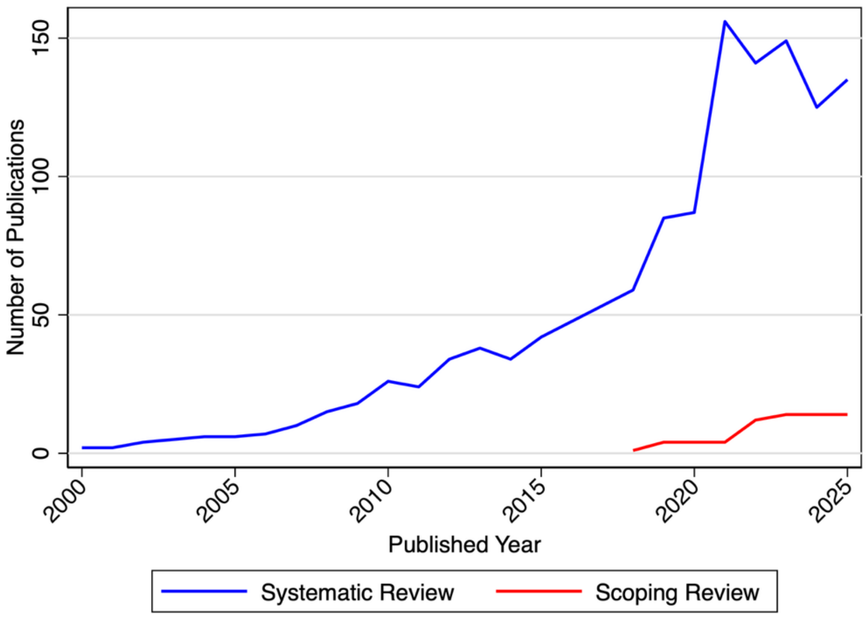

1.3

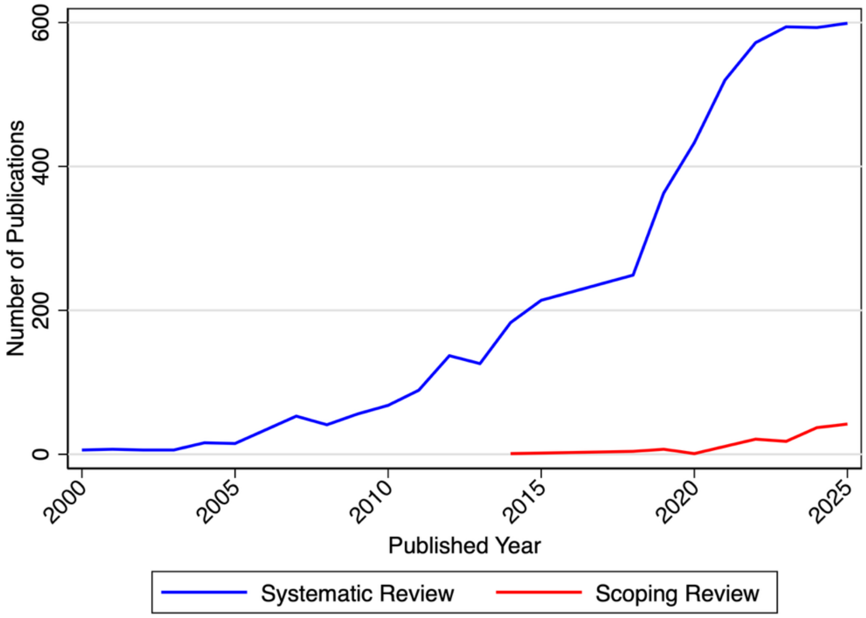

Number of Publications
0
200
400
600
2000
2005
2010
2015
2020
2025
Published Year
Systematic Review
Scoping Review

1.4

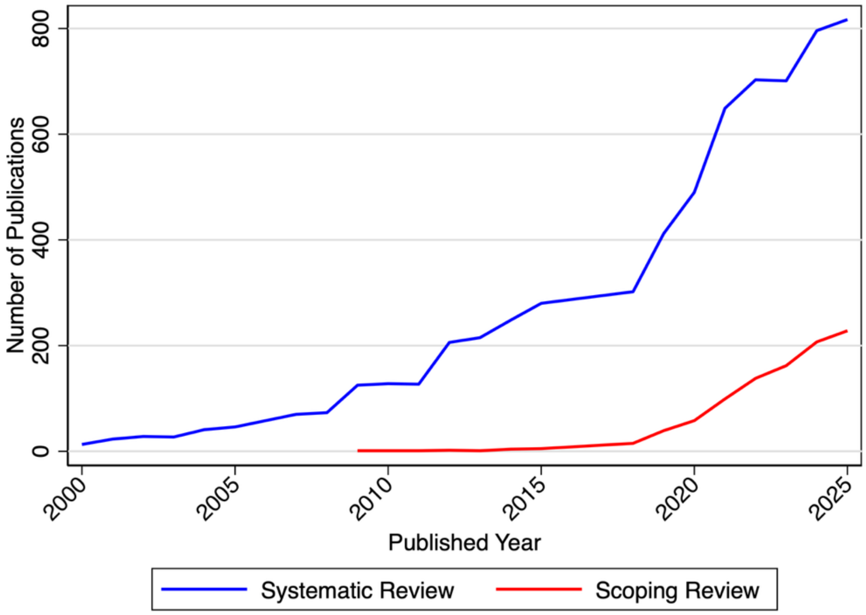

Number of Publications
0
200
400
600
800
2000
2005
2010
2015
2020
2025
Published Year
Systematic Review
Scoping Review

1.5

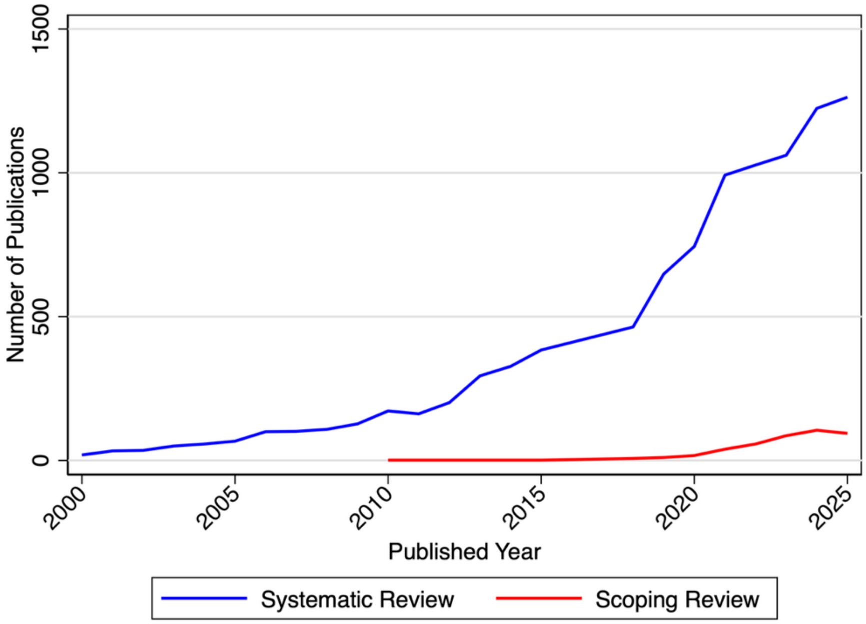

Number of Publications
1500
1000
500
0
2000
2005
2010
2015
2020
2025
Published Year
Systematic Review
Scoping Review

Figure S2. Average Time (Months) Between Search Date and Publication Date by Review Type, within **2.1** Medical Oncology **2.2** Radiation Oncology **2.3** Surgical Oncology **2.4** Supportive Oncology **2.5** Multiple Subspecialties

2.1

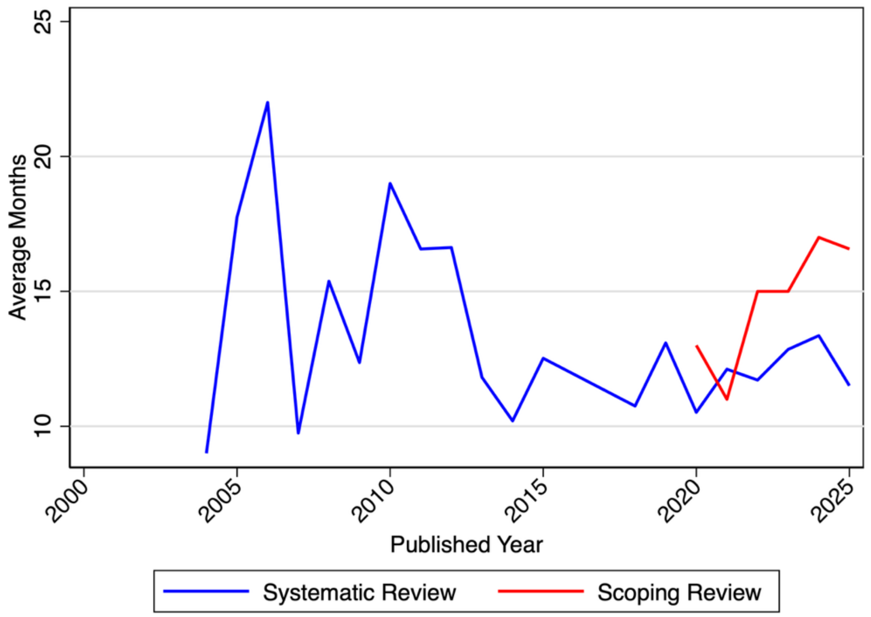

2.2

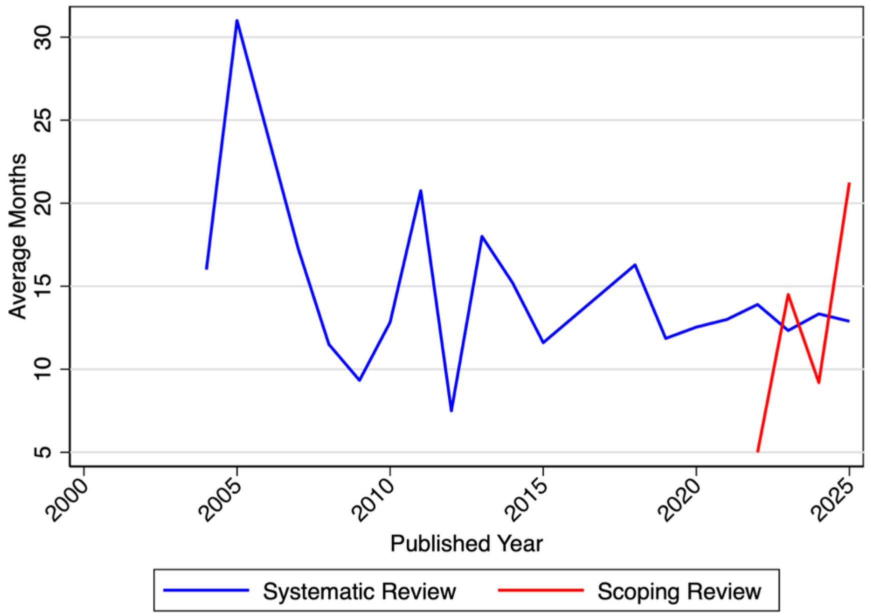

2.3

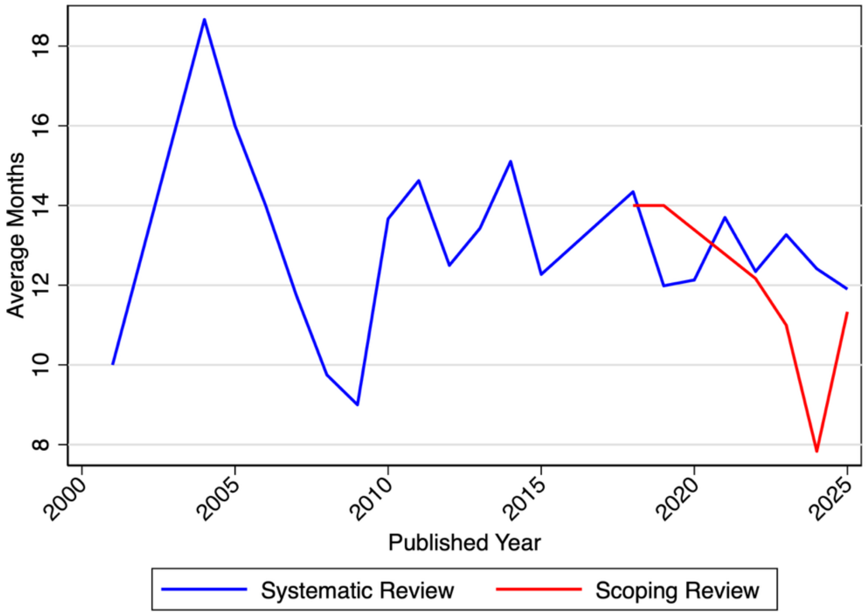

Average Months
18
16
14
12
10
8
2000
2005
2010
2015
2020
2025
Published Year
Systematic Review
Scoping Review

2.4

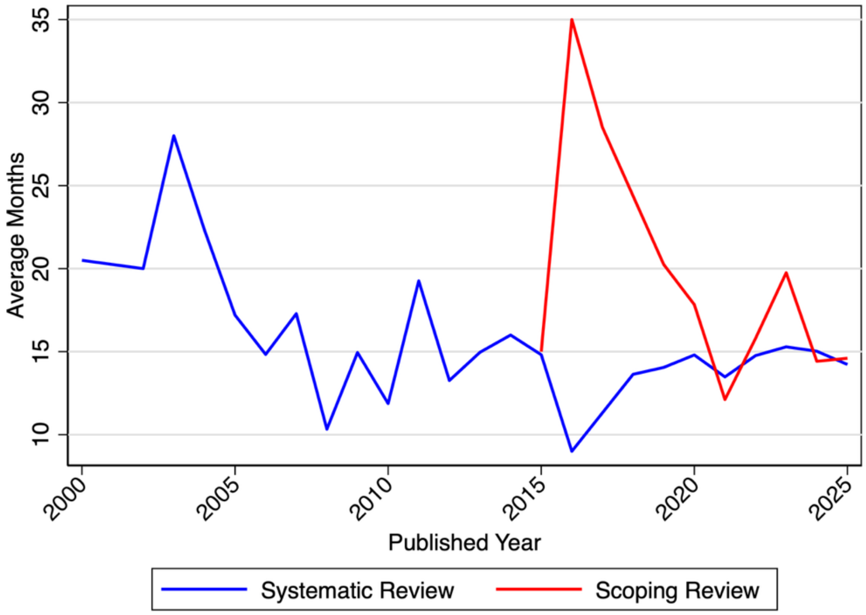

Average Months
35
30
25
20
15
10
2000
2005
2010
2015
2020
2025
Published Year
Systematic Review
Scoping Review

2.5

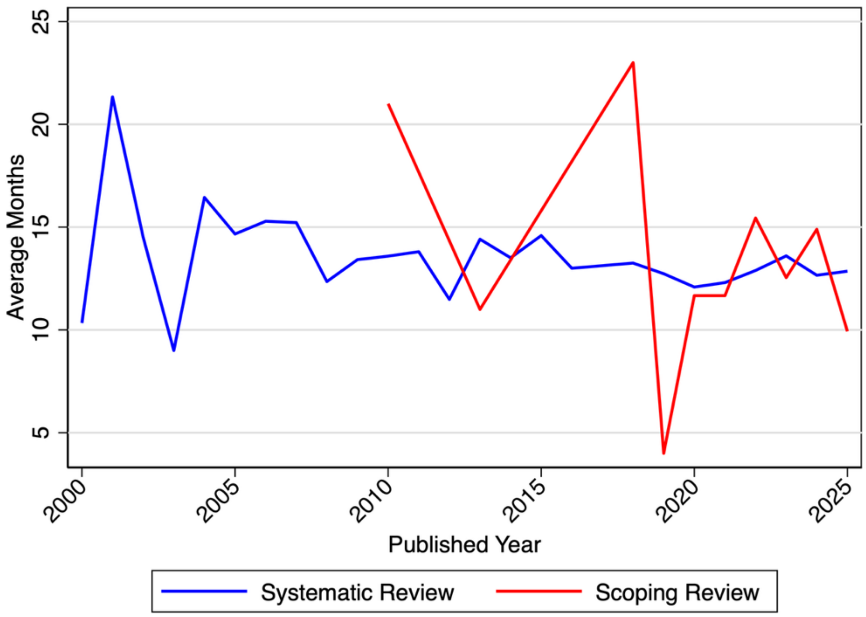
Average Months
25
20
15
10
5
2000
2005
2010
2015
2020
2025
Published Year
Systematic Review
Scoping Review
