# Supplementary material for: Trends in Volume, Timeliness, and Reporting Quality of Oncology Systematic and Scoping Reviews: A 25-Year Analysis
Source: Curr Oncol. 2026 Sep 21;33(9):571. doi: 10.3390/curroncol33090571 (PMC13604965; doi:10.3390/curroncol33090571)
Supplement: Supplementary file 1 [file curroncol-33-00571-s001.zip › File S1_Search Strategy.pdf]

Supplementary File S1. Search Strategies

Medline (PubMed):

Systematic Review Search Strategy:

(umbrella review*[tw] OR systematic review[pt] OR systematic review as topic[mh] OR systematic review*[tw])

AND

(cancer*[tw] OR neoplasms[mh] OR neoplas*[tw] OR oncolog*[tw] OR tumor*[tw] OR tumour*[tw])

Scoping Review Search Strategy:

(scoping review[pt] OR scoping review as topic[mh] OR scop* review*[tw])

AND

(cancer*[tw] OR neoplasms[mh] OR neoplas*[tw] OR oncolog*[tw] OR tumor*[tw] OR tumour*[tw])

Ovid EMBASE & Cochrane Library

Systematic Review Search Strategy:

(exp "systematic review (topic)"/ or exp "systematic review"/ or umbrella review*.mp. or exp "umbrella review"/ or systematic review*.mp.)

and

(cancer*.mp. or neoplasms.mp. or exp neoplasm/ or exp oncology/ or oncolog*.mp. or tumor*.mp. or tumour*.mp.)

Scoping Review Search Strategy:

(exp "scoping review"/ or exp "scoping review (topic)"/ or scop* review*.mp.)

and

(cancer*.mp. or neoplasms.mp. or exp neoplasm/ or exp oncology/ or oncolog*.mp. or tumor*.mp. or tumour*.mp.)
